# Food-Grade Microemulsion for High-Loading Octacosanol: Formulation Optimization, Characterization, and Biological Evaluation

**DOI:** 10.3390/foods15122154

**Published:** 2026-06-15

**Authors:** Jiayi Lin, Shengang Yao, Lanlan Li, Wanrong Li, Fangxue Hang, Kai Li, Caifeng Xie

**Affiliations:** College of Light Industry and Food Engineering, Guangxi University, Nanning 530004, China; linjiayi0923@126.com (J.L.); yaoshengang@163.com (S.Y.); lillian113148@gmail.com (L.L.); lwr9812a@163.com (W.L.); hangfx@163.com (F.H.); gxlikai@gxu.edu.cn (K.L.)

**Keywords:** octacosanol, microemulsion, functional beverage, stability, in vitro digestion, biocompatibility

## Abstract

Octacosanol (OCT) is a natural bioactive compound with multiple physiological activities. However, its poor aqueous solubility limits its application in functional beverages, and existing delivery systems suffer from low loading and excessive emulsifier use. This study aimed to develop a food-grade OCT-loaded microemulsion (OCT-ME) with high loading capacity. The formulation was optimized via pseudo-ternary phase diagram analysis combined with particle size and polydispersity index (PDI) measurements, and the characterization and biocompatibility of the optimized OCT-ME were systematically evaluated. The optimal formulation (*w*/*w*) consisted of 2.4% corn oil, 16.2% mixed emulsifiers (Tween 80/Span 80, HLB = 13), 5.4% 1,2-propanediol (Km = 3:1), and 75.0% deionized water, achieving a high OCT loading capacity of 1.0% (*w*/*w*). The resulting OCT-ME displayed a uniform particle size of 10.37 nm with a low PDI and exhibited excellent stability, favorable gastric OCT protection, and superior biocompatibility (cell viability > 90% at 5–25 μg/mL). This work addresses the key limitations of existing OCT delivery systems, providing theoretical support for the efficient solubilization and delivery of OCT in functional beverages.

## 1. Introduction

With increasing global health awareness, the functional food market has expanded rapidly and is projected to reach USD 595.49 billion by 2033 from USD 359.81 billion in 2025 [1]. In China, the market size of health and functional foods has grown robustly, surging from RMB 222.7 billion in 2019 to RMB 298.9 billion in 2022 and is expected to maintain its growth in the future [2]. Functional beverages stand out as the fastest-growing segment, owing to their portability, ready-to-consume convenience, high bioavailability, and favorable sensory appeal. These attributes enable them to meet the health-oriented demands of modern fast-paced lifestyles [3].

The health benefits of functional beverages mainly derive from their bioactive components. Growing public awareness of green and safe consumption has driven consumer preference for functional beverages containing natural rather than synthetic additives. Plant-derived extracts and naturally derived functional ingredients dominate among bioactive substances nowadays [4]. Octacosanol (OCT), a highly hydrophobic long-chain aliphatic alcohol abundant in the plant cuticular and wax layers [5], possesses diverse bioactivities including lipid metabolism regulation, antioxidant, anti-fatigue, and anti-inflammatory properties; improved exercise endurance; and enhanced cardiovascular health [6]. Consequently, OCT has been widely utilized in functional foods and pharmaceuticals. However, its extremely poor water solubility severely hinders its application in aqueous functional beverages.

An oil-in-water microemulsion (O/W ME) is a thermodynamically stable nanodispersed system that can effectively enhance the aqueous solubility and bioavailability of poorly water-soluble bioactive compounds [7]. It has been successfully applied to the aqueous delivery of various lipophilic functional ingredients, including curcumin [8], α-tocopherol [9], and coenzyme Q10 [10]. In recent years, growing research efforts have been focused on the development of OCT-loaded microemulsion (OCT-ME) for the formulation of OCT-based functional beverages. Jia et al. [11] successfully fabricated an OCT-ME employing ethyl acetate (5.50%, *w*/*w*) as both the oil phase and co-emulsifier, with polyoxyethylene hydrogenated castor oil (5.56%, *w*/*w*) serving as the emulsifier, achieving an OCT loading capacity of 0.55% (*w*/*w*). However, the incorporation of ethyl acetate, a non-food-grade organic solvent, severely limits the practical application of such OCT-MEs in the food and beverage industry. To address this issue, Zhu et al. [12] developed a food-grade OCT-ME composed of olive oil, Tween 80, and glycerol, which achieved an OCT loading capacity of 0.1% (*w*/*w*). Although this OCT-ME demonstrated excellent food safety, its practical application was restricted by the excessively high total dosage of emulsifier and co-emulsifier (31.67%, *w*/*w*), and extremely low OCT loading capacity. Wang et al. [13] fabricated an OCT-ME using corn oil as the oil phase and Tween 80 as the emulsifier (4.50%, *w*/*w*); however, the OCT loading capacity remains low at 0.1% (*w*/*w*). Collectively, these existing studies exhibit prominent limitations, highlighting the urgent need to develop food-grade OCT-MEs with high OCT loading capacity to promote the industrial application of OCT-based functional beverages.

The loading capacity of OCT in MEs is primarily governed by its solubility in the oil phase. Specifically, plant oils rich in long-chain triglycerides (LCTs) possess large hydrophobic cores and exhibit robust hydrophobic interactions, enabling the formation of sufficiently large micelles to encapsulate OCT, thus conferring excellent OCT solubilization capacity. Plant oils containing C16-C18 fatty acids further enhance this capacity through hydrophobic interaction with OCT. Consequently, they have been widely adopted as oil components in OCT-ME formulations [14,15]. Emulsifiers and co-emulsifiers play multiple roles in O/W ME. They reduce the interfacial tension and strengthen the interfacial film, thereby improving the thermodynamic and kinetic stability of O/W ME. Additionally, they can enhance the solubility of hydrophobic bioactive compounds, which directly enhances the bioactive loading capacity of O/W MEs. An appropriate hydrophilic–lipophilic balance (HLB) value of emulsifiers is crucial for fabricating high-quality O/W MEs [16]. Emulsifiers with HLB values of 8–18 are suitable for constructing O/W ME [17]. As a food-grade emulsifier, Tween is widely adopted in O/W MEs owing to its non-toxicity and favorable biocompatibility. It exhibits dual functionality: the hydrophilic segments enhance the loading of bioactive compounds, while the polyoxyethylene moiety provides steric stabilization [18,19].

However, O/W MEs prepared with a single emulsifier generally exhibit poor stability [20]. Consequently, mixed emulsifier systems, especially Tween-Span blends, have attracted increasing attention. Their molecular synergism, including electrostatic interactions of polar head groups and complementary hydrophobic chains, reduces interfacial tension and critical micelle concentration (CMC) [21,22] and thus improves the stability of O/W MEs. Therefore, Tween and Span are widely employed as mixed emulsifiers in food-grade O/W MEs [23].

Low-molecular-weight co-emulsifiers are also indispensable components of O/W MEs. They further reduce interfacial tension and enhance the fluidity of interfacial films, broadening the microemulsion formation region and boosting the solubility of bioactive compounds [24]. Pseudoternary phase diagrams are widely adopted to optimize O/W ME formulations. Nevertheless, most existing studies on OCT-ME focus on preparation methods and in vivo bioactivity evaluation, while systematic formulation optimization remains insufficient. Specifically, the effects of long-chain OCT on the stability of O/W ME have not been fully elucidated [25,26].

This study aims to develop a food-grade OCT-ME with low emulsifier/co-emulsifier content and high OCT loading. First, the solubility of OCT in various oils was systematically compared to screen for the most suitable oil phase. Subsequently, pseudo-ternary phase diagrams were constructed, and the influence of OCT incorporation on the stability of the O/W ME was investigated to optimize the formulation of the O/W ME, including the oil phase, emulsifiers, and co-emulsifiers. The resulting OCT-ME was comprehensively characterized and evaluated in terms of its physicochemical properties, stability, and bioactivity. The findings are anticipated to provide a theoretical foundation for the development of OCT-based functional beverages.

## 2. Materials and Methods

### 2.1. Raw Materials and Reagents

OCT (purity ≥ 90%) was purchased from Xi’an Shouhe Biotechnology Co., Ltd. (Xi’an, China). Corn oil, soybean oil, peanut oil, olive oil, and sunflower oil were obtained from COFCO Group Co., Ltd. (Beijing, China). Tween 20, Tween 40, Tween 80, and Span 80 were supplied by Xuzhou Kunrong Food Ingredients Co., Ltd. (Xuzhou, China). Glycerol, 1,2-propanediol, and absolute ethanol were purchased from Lianyungang Xin’ai Food Technology Co., Ltd. (Lianyungang, China). All these agents were food-grade. Chromatographic-grade chloroform was obtained from Nuoshi Technology Co., Ltd. (Shenzhen, China). High-purity OCT (purity ≥ 98%) was purchased from Shanghai Yuanye Bio-Technology Co., Ltd. (Shanghai, China). All other chemicals were of analytical grade.

### 2.2. Solubility of OCT in Different Oil Phases

Excess OCT (0.1 g) was added to each oil phase (10 mL), followed by thorough vortex mixing for 1 min using a vortex shaker (LP Vortex Mixer, Seoul, Republic of Korea). The mixture was then sonicated at 500 W for 30 min in an ultrasonic cleaner (KQ-500DE, Kunshan, China). Subsequently, the samples were incubated at 37 °C for 48 h in a constant-temperature shaking incubator (ZQZY-85CN, Shanghai, China) to achieve equilibrium. After equilibrium, the samples were centrifuged at 10,000 rpm for 20 min using a desktop centrifuge (CenLee18R, Tokyo, Japan). The resulting supernatant was collected, extracted with chromatographic-grade chloroform, and filtered through a 0.22 μm organic phase filter. OCT content was quantified via a gas-chromatography–mass-spectrometry (GC-MS, 8860-5977C, Santa Clara, CA, USA). The oil phases with high OCT solubility were selected as oil phase carriers for subsequent microemulsion construction.

### 2.3. Construction of Pseudo-Ternary Phase Diagrams

Pseudo-ternary phase diagrams were constructed using the water titration method to systematically evaluate the effects of different oil phases, emulsifiers, co-emulsifiers, HLB values, and Km values (mass ratio of emulsifier to co-emulsifier) on O/W ME formation. For each variable investigated, all other parameters were held constant. Emulsifier and co-emulsifier were mixed at a fixed Km value to obtain the surfactant/co-surfactant mixture (Smix). Smix was then combined with the oil phase at various mass ratios (Smix:oil = 9:1 to 1:9, *w*/*w*), and each mixture was placed in a constant-temperature water-bath magnetic stirrer (ZNCL-S-10G, Shanghai, China). The mixture was stirred at 500 rpm for 10 min at 80 °C to ensure homogeneous mixing. While maintaining 80 °C, 80 °C ultrapure water was added dropwise to each mixture, and the mass of water required to reach the turbidity-to-clarity transition point (critical point of O/W ME formation) was recorded. The mass percentages of each phase were then calculated. Pseudo-ternary phase diagrams were constructed using Origin 2022 software, and the area percentage of the O/W ME region was quantified using Photoshop 2017 software. A schematic diagram displaying the complete phase distribution is shown in Figure S1.

### 2.4. Optimization of O/W ME Formulation

Based on the pseudo-ternary phase diagrams, the mass ratio of oil phase to Smix was fixed at 1:9 (*w*/*w*) to obtain transparent microemulsions for most formulations. OCT-ME loaded with 1.0% (*w*/*w*) OCT and blank microemulsions were fabricated under varied oil phases, emulsifier, co-emulsifier, HLB and Km values. For OCT-ME preparation, OCT was pre-dissolved in the oil phase before blending with Smix, followed by stirring at 500 rpm and 80 °C for 10 min. Preheated ultrapure water (80 °C) was supplemented dropwise at constant temperature until water accounted for 75% (*w*/*w*) of the total system, and then the mixture was continuously stirred for another 30 min. Blank samples were synthesized via the same protocol. Particle size and polydispersity index (PDI) were measured with a Malvern Zetasizer Nano ZS90 (Malvern, UK). The optimum formulation was screened by comprehensive comparison of O/W ME area fraction, particle size, PDI and macroscopic morphology among all groups.

### 2.5. OCT Loading Capacity of O/W ME

Under the optimized formulation system, O/W MEs with various OCT loading concentrations (0.1–1.2%, *w*/*w*) were prepared. The particle size and PDI of each formulation were measured, with a particle size ≤ 30 nm and a PDI < 0.5 adopted as stability criteria to evaluate the maximum OCT loading capacity.

### 2.6. Characterization of OCT-ME

#### 2.6.1. Droplet Size and PDI

After being diluted 5-fold with ultrapure water, the samples were analyzed for particle size and PDI at room temperature using a nanoparticle size analyzer (Malvern Zetasizer Nano ZS90, Malvern, UK). The equilibration time was set to 120 s at a detection angle of 90°, and each sample was measured in triplicate.

#### 2.6.2. Transmission Electron Microscopy (TEM)

After being diluted to an appropriate volume with ultrapure water, an aliquot of the OCT-ME solution was placed onto a copper grid and left to stand for 2 min. The sample was then negatively stained with 2% (*w*/*v*) phosphotungstic acid for an additional 2 min, and excess liquid was blotted off with filter paper. Following air-drying, the microstructure of OCT-ME was observed using a transmission electron microscope (TEM, HT7700, Tokyo, Japan).

#### 2.6.3. OCT-ME Type Determination

The type of OCT-ME was identified by electrical conductivity measurements and dye solubilization assays. OCT-ME samples with water contents ranging from 0% to 90% (*w*/*w*) were prepared, and conductivity was measured with a conductivity meter (FE38, Shanghai, China). In addition, water-soluble methylene blue (1 g/L) and oil-soluble Sudan red (1 g/L) were used to distinguish ME type by their diffusion behavior.

#### 2.6.4. Viscosity Measurement

The viscosity of OCT-MEs was measured using a rotational viscometer (NDJ-8S, Shanghai, China) equipped with a No. 3 spindle at 25 ± 2 °C.

#### 2.6.5. Fourier Transform Infrared (FT-IR) Spectroscopy

FT-IR spectroscopy (TENSOR, Bruker Optics, Ettlingen, Germany) was used to characterize OCT-free O/W MEs and OCT-loaded O/W MEs at concentrations of 5 and 10 mg/mL. Comparative spectral analyses were performed among pure OCT, corn oil, Tween 80, Span 80, propylene glycol, deionized water, and the prepared OCT-MEs. The spectra were recorded over a scanning range of 650–4000 cm^−1^ at a resolution of 4 cm^−1^.

### 2.7. Stability of OCT-ME

The stability of OCT-ME for functional beverage applications and storage was evaluated by environmental stress testing and long-term storage studies. Phase separation or turbidity was used as a qualitative stability indicator, while changes in average particle size and PDI served as quantitative parameters.

#### 2.7.1. Environmental Stress Stability Evaluation

Stability was evaluated under various stress conditions: centrifugation (2000–10,000 rpm for 10 min), temperature cycling (−20 to 80 °C for 30 min), pH 1.0–11.0, and NaCl concentrations of 0.1–2.0 mol/L. These conditions were applied to assess its resistance to external physical disturbances and fluctuations in chemical environments.

#### 2.7.2. Storage Stability Evaluation

The storage stability of OCT-ME was evaluated under refrigerated (4 °C) and ambient (25 °C) conditions. Samples were collected on days 1, 7, 14, 21, 30, 60, 120, 180, 240, 300, and 360 to monitor appearance variations and evaluated long-term storage stability.

### 2.8. In Vitro Digestion

A static in vitro digestion model was established according to the method described by Minekus et al. [27]. Electrolyte stock solutions of simulated saliva fluid (SSF), simulated gastric fluid (SGF), and simulated intestinal fluid (SIF) were prepared, with their detailed compositions and concentrations listed in Table S1. The experiment was performed in three consecutive phases (oral, gastric, intestinal) at a constant temperature of 37 °C. Two parallel groups were designed to compare OCT digestive release profiles: an experimental group of optimally formulated OCT-ME, and a control group of OCT suspension. Both groups had an OCT concentration of 1.0% (*w*/*w*), with the suspension stabilized by 1% (*w*/*w*) sodium carboxymethyl cellulose.

A 5 mL aliquot of OCT-ME was mixed with 4 mL of pre-warmed SSF (37 °C). Calcium chloride (CaCl_2_) was added to a final concentration of 0.75 mM, and the volume was adjusted to 10 mL with ultrapure water, followed by incubation at 37 °C with shaking at 150 rpm for 2 min. The oral digesta were mixed with 8 mL of SGF, and pepsin (2000 U/mL), lipase (60 U/mL), and CaCl_2_ (0.075 mM) were added; the total volume was adjusted to 20 mL with ultrapure water, and the pH was adjusted to 3.0 with 1 M HCl, and then the mixture was incubated at 37 °C with shaking at 150 rpm for 2 h. The gastric digesta were mixed with 16 mL of SIF, and trypsin (100 U/mL), lipase (2000 U/mL), bile salts (10 mg/mL), and CaCl_2_ (0.3 mM) were added; the total volume was adjusted to 40 mL with ultrapure water, and the pH was adjusted to 7.0 with 1 M NaOH, then incubated at 37 °C with shaking at 150 rpm for 2 h.

Samples were collected at the end of the oral, gastric, and intestinal digestion phases. To terminate enzymatic activity, each sample was immediately heated in a boiling water bath for 10 min and then cooled in an ice bath. Finally, the samples were centrifuged at 8000 rpm for 15 min, and the supernatant was carefully collected for subsequent analysis.

### 2.9. Biocompatibility Evaluation

Caco-2 cells were used as an in vitro model of intestinal absorption. Cells were cultured at 37 °C in a humidified atmosphere with 5% CO_2_.

#### 2.9.1. Cell Viability Assay

Caco-2 cells in the logarithmic growth phase were seeded into 96-well plates at a density of 8 × 10^3^ cells/well. After overnight incubation, control medium, blank MEs, and OCT-MEs (5–50 μg/mL) were added to the wells, respectively, and cells were incubated for a further 24 h. Cell viability was determined using the CCK-8 assay, with absorbance measured at 450 nm using a microplate reader(SPARK 10M, Tecan Trading AG, Männedorf, Switzerland).

#### 2.9.2. Live/Dead Cell Staining

Caco-2 cells were seeded into confocal dishes at a density of 2 × 10^5^ cells/dish and cultured overnight under standard conditions. The cells were treated with control medium or OCT-ME at concentrations of 25 and 50 μg/mL, followed by further incubation for 24 h. Cells were stained with Calcein-AM/propidium iodide (PI). Cells were stained in the dark for 15 min, rinsed with phosphate-buffered saline (PBS), and observed under a fluorescence microscope.

### 2.10. Statistical Analysis

All experiments were performed in triplicate, and results are expressed as mean ± standard deviation. Statistical analyses were performed using one-way analysis of variance followed by Tukey’s test (SPSS 22.0). Differences were considered statistically significant at *p* < 0.05.

## 3. Results and Discussion

### 3.1. Analysis of OCT Solubility Behavior in Oil Phase and Oil Phase Selection

The selection of an appropriate oil phase is crucial for the preparation of OCT-ME, as it directly impacts the solubility of OCT and consequently affects the loading capacity and stability of OCT-ME. As shown in Table 1, the solubility of OCT varied significantly among different oil phases, with the highest solubility (49.07 ± 0.07 mg/L) observed in peanut oil. This outstanding solubility behavior was primarily attributed to the high content of unsaturated fatty acids in peanut oil, particularly oleic acid (C18:1) [28]. Notably, the linear molecular arrangement and cis double-bond-induced molecular curvature of oleic acid enhance the fluidity of the oil phase and strengthen hydrophobic interactions, which facilitates the stable formation of OCT-ME and enhances OCT solubilization [29,30]. Furthermore, the amphiphilic structure of endogenous phospholipids in peanut oil enables the oil phase to be adsorbed and aligned at the oil–water interface, resulting in enhancement in interfacial stability of O/W MEs and further increased OCT-loading capacity [31].

The OCT solubility in coconut oil was 46.32 ± 0.07 mg/L, ranking second only to that in peanut oil. This relatively high solubility can be attributed to the high content of medium-chain fatty acids (e.g., lauric acid, C12:0) in coconut oil, whose strong polarity and potential dipole–dipole interactions with OCT molecules might enhance OCT solubility. However, the high proportion of saturated fatty acids promotes crystallization even at room temperature. This can lead to an inhomogeneous distribution of OCT within the oil phase, thereby compromising the physical stability of OCT-ME. Consequently, coconut oil was excluded from subsequent selection of oil phases for OCT-ME [32,33].

The solubility of OCT in corn oil and soybean oil was 39.67 mg/L and 37.67 mg/L, respectively, both of which were significantly lower than that in peanut oil. This difference is closely associated with their distinct fatty acid compositions: both oils are rich in linoleic acid (C18:2). Such polyunsaturated fatty acids may result in weaker hydrophobic interactions between oil molecules and OCT compared to those of oleic acid, thereby reducing the overall solubilization capacity for OCT [34].

Notably, OCT exhibited the lowest solubility in olive oil despite its high oleic acid content. A key contributing factor is the abundance of natural polar components in olive oil, such as polyphenols, squalene, and vitamin E. These highly polar compounds possess strong polarity and robust intermolecular interactions, which may inhibit the effective dispersion and solubilization of OCT by competing with the oil phase for solubilization sites or modifying the oil-phase microenvironment [35,36].

Based on the above analysis, peanut oil, corn oil, and soybean oil were selected as oil phases for further investigation of OCT-ME. While corn oil and soybean oil exhibited relatively lower OCT solubility, they offer significant industrial advantages due to their low cost and wide availability. More importantly, polyunsaturated fatty acids (e.g., linoleic acid) can promote the formation of mixed micelles and enhance the OCT solubilization capacity by increasing lipid layer fluidity and interfacial film disorder [37]. Therefore, the inclusion of corn oil and soybean oil in this study is expected to provide valuable insights for optimizing O/W ME formulations.

### 3.2. Construction and Formulation Optimization of OCT-ME

In the present study, the formulation of OCT-ME was optimized using a combined approach consisting of pseudo-ternary phase diagram construction and dynamic light scattering (DLS) analysis. As a classical tool for formulating O/W ME, the pseudo-ternary phase diagram allows direct visualization of phase transitions and phase boundaries between the oil phase, Smix, and aqueous phase. This approach helps identify microemulsion regions with excellent physical stability and determine optimal component proportions. However, OCT is a water-insoluble solid at ambient temperature, which hinders direct observation of the phase behavior among the oil phase, Smix, and aqueous phase. Accordingly, pseudo-ternary phase diagrams were constructed using drug-free O/W MEs [38]. Upon incorporation of OCT, interactions between OCT and the formulation components may alter the system’s properties and induce instability such as particle aggregation and phase separation even in formulations initially judged stable by phase diagram analysis [39,40]. Thus, relying solely on the pseudo-ternary phase diagram is insufficient to predict the stability of drug-loaded microemulsions; particle size and PDI following OCT loading must also be considered. Integrating pseudo-ternary phase diagrams with DLS analysis enables identification of formulations exhibiting both a large stable region and favorable colloidal properties (small particle size and low PDI) after drug loading, thereby ensuring effective OCT solubilization in a physically stable O/W ME system [41].

#### 3.2.1. Determination of the Oil Phase

**Figure 1:**
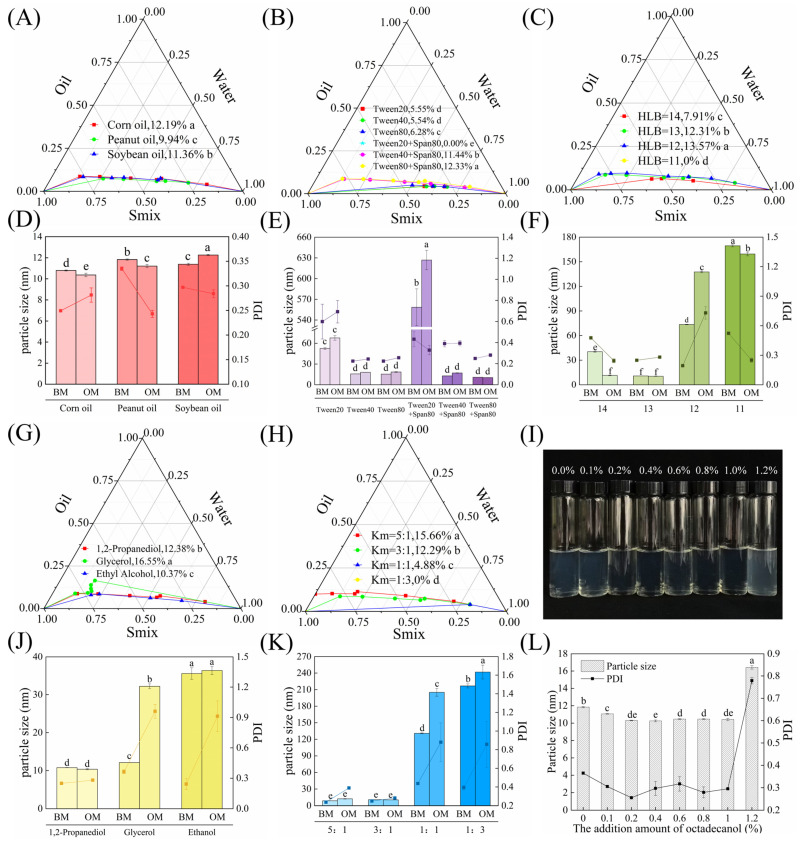
Pseudo-ternary phase diagrams of the microemulsion systems under different oil phases (**A**), emulsifiers (**B**), hydrophilic–lipophilic balance (HLB) values (**C**), co-emulsifiers (**G**), and emulsifier-to-co-emulsifier mass ratio (Km) values (**H**); particle size and polydispersity index (PDI) of microemulsions before and after octacosanol (OCT) loading under different oil phases (**D**), surfactants (**E**), HLB values (**F**), co-surfactants (**J**), and Km values (**K**); and appearance (**I**), particle size, and PDI (**L**) of OCT-loaded microemulsion (OCT-ME) at different OCT loadings. Different lowercase letters within the same group indicate significant differences (*p* < 0.05).

Analysis of the pseudo-ternary phase diagrams (Figure 1A) revealed that the area percentage of the stable microemulsion region was 12.19 ± 0.01% for corn oil, which was significantly larger than that of peanut oil (9.94 ± 0.17%) and that of soybean oil (11.36 ± 0.11%), indicating its superior emulsifying capacity and stability. This phenomenon was attributed to corn oil’s abundant nonpolar components, which exhibit strong compatibility with emulsifier hydrophobic moieties, are effectively inserted into the oil–water interfacial film, lower interfacial tension, and enlarge the stable region [42,43].

To further evaluate the emulsifying efficiency of different oil phases and the impact of OCT loading, the variations in particle size and PDI before and after OCT addition were compared (Figure 1D). In the absence of OCT, the microemulsions formulated with corn oil exhibited the smallest mean particle size (10.79 ± 0.05 nm) and lowest PDI (0.249 ± 0.002) compared to those prepared with peanut oil (particle size 11.83 ± 0.06 nm, PDI 0.335 ± 0.003) and soybean oil (particle size 11.38 ± 0.10 nm, PDI 0.297 ± 0.002), suggesting a more uniform droplet distribution. Upon OCT incorporation, the particle size changes among the systems diverged markedly. Specifically, the particle size of the soybean oil microemulsions increased significantly to 12.26 ± 0.06 nm (PDI 0.284 ± 0.008), demonstrating that OCT incorporation compromised the structural integrity of these systems. In contrast, the particle sizes of corn and peanut oil microemulsions were reduced to 10.37 ± 0.17 nm (0.281 ± 0.014) and 11.21 ± 0.15 nm (PDI 0.243 ± 0.007), respectively. Despite the particle size reduction in peanut oil after OCT loading, its overall performance remained suboptimal, due to its narrow stable region and larger final size than corn oil. Meanwhile, the corn oil system maintained the smallest size and a relatively low PDI, demonstrating excellent compatibility with OCT. This was primarily attributed to its nonpolar components preferentially localizing in the micellar core and forming a dense interfacial film with emulsifiers, which allowed OCT to be uniformly dispersed in the oil phase without interfacial accumulation, thus effectively preserving the structural stability of the microemulsion. In contrast, soybean oil is rich in polar components that tend to segregate at the oil–water interface; the introduction of OCT readily disrupts the interfacial film structure, weakens its stabilizing effect, and consequently leads to droplet coalescence and increased particle size [44,45]. Taken together, corn oil exhibited the optimal overall performance in terms of microemulsion formation capability, structural stability, and OCT compatibility. Therefore, corn oil was selected as the optimal oil phase for OCT-ME.

#### 3.2.2. Determination of Emulsifier

As illustrated in Figure 1B, the stable microemulsion regions generated by most mixed emulsifiers are significantly larger than those of single emulsifiers, showing superior emulsifying capacity. This observation can be attributed to the insufficient compatibility between the high HLB values of single emulsifiers and the moderately polar corn oil. By contrast, mixed emulsifier systems can optimize the overall HLB value and molecular compatibility through synergistic interactions, effectively reducing interfacial tension to promote microemulsion formation and enlarge the stable region [46,47]. Among all combinations, the Tween 80/Span 80 pair exhibited the best emulsifying performance, producing the largest microemulsion area (12.33 ± 0.01%). This superiority arises primarily from their identical alkyl tails but distinctly different hydrophobic headgroup sizes, creating an optimal synergistic effect [48]. Moreover, the carbon–carbon double bond in the lipophilic chain of Tween 80 provides higher flexibility and hydrophilicity relative to saturated linear alkyl chains, further enhancing its emulsifying properties [49].

To further evaluate the emulsifying efficiency of different emulsifier systems and the impact of OCT loading, the variations in particle size and PDI before and after OCT addition were compared (Figure 1E). In the absence of OCT, microemulsions stabilized by the Tween 80/Span 80 blend not only displayed the largest microemulsion region but also exhibited the smallest mean particle size (10.79 ± 0.05 nm, PDI = 0.249 ± 0.002). Upon OCT incorporation, the systems showed divergent changes in particle size. Specifically, the particle sizes of single-emulsifier systems and the Tween 40/Span 80 blend increased to varying degrees, indicating that the OCT loading disrupted the original interfacial film equilibrium and reduced their resistance to droplet coalescence. Meanwhile, the Tween 20/Span 80 system barely formed a stable microemulsion, with its particle size surging to 626.87 ± 14.07 nm (PDI = 0.328 ± 0.044) after OCT addition. In contrast, the particle size of the Tween 80/Span 80 microemulsion did not increase but instead decreased slightly to 10.37 ± 0.17 nm. Meanwhile, it maintained a relatively low PDI (0.281 ± 0.014), demonstrating excellent coalescence resistance and good compatibility with OCT. This phenomenon could be attributed to the strong compatibility between OCT and the C18 hydrophobic tail chains of both Tween 80 and Span 80. Furthermore, the unsaturated bond in Tween 80 enabled its molecules to align more parallel to the hydrophobic interface, thereby effectively preserving or even enhancing interfacial film stability and inhibiting droplet coalescence and maintaining a small particle size [50]. Taken together, the Tween 80/Span 80 blend exhibited the optimal overall performance in terms of microemulsion formation capacity, structural stability, and OCT compatibility. Therefore, it was selected as the optimal emulsifier for OCT-ME.

#### 3.2.3. Determination of the HLB Value

The HLB value can be flexibly modulated by adjusting the blending ratio of Tween 80 and Span 80. Given the moderate polarity of corn oil, an HLB range of 11–14 was selected for systematic screening. Analysis of the pseudo-ternary phase diagrams (Figure 1C) revealed marked differences in stable microemulsion regions among various HLB values. The system with HLB = 12 exhibited the largest stable microemulsion area (13.57 ± 0.03%), followed by those at HLB = 13 (12.31 ± 0.07%) and HLB = 14 (7.91 ± 0.08%), whereas no stable microemulsion domain was observed at HLB = 11. These results indicated that the blended emulsifiers at HLB = 12 achieved optimal polarity matching with corn oil. At this HLB value, emulsifier molecules are closely packed at the oil–water interface, forming a stronger interfacial film and generating greater inter-droplet repulsion, thereby conferring enhanced stability to the microemulsion [51]. However, at HLB = 13 and 14, the enhanced hydrophilicity of the emulsifier blend weakened its interaction with the oil phase, thereby hindering micellization and narrowing the stable microemulsion region. In contrast, at HLB = 11, the emulsifier system exhibited insufficient hydrophilicity, which impeded hydrogen bonding with the aqueous phase, reduced the water-holding capacity of the microemulsion, and consequently prevented the formation of a stable microemulsion [52].

As illustrated in Figure 1F, OCT loading exerted a significant impact on the stability of microemulsions prepared at different HLB values. In the OCT-free systems, the system at HLB = 13 exhibited the smallest mean particle size (10.79 ± 0.05 nm) and a relatively low PDI (0.249 ± 0.002), indicating that droplets were finely and uniformly distributed. Although the HLB = 12 system displayed the largest region in the pseudo-ternary phase diagram, its mean particle size was relatively large (73.48 ± 0.34 nm, PDI = 0.194 ± 0.004), which is unfavorable for long-term storage. Upon OCT incorporation, divergent changes in particle size were observed. Specifically, the particle size of the HLB = 12 system increased sharply to 137.57 ± 0.98 nm, accompanied by an increase in PDI to 0.730 ± 0.063. This demonstrated that incorporation of OCT, a long-chain fatty alcohol, altered the overall polarity of the oil phase, resulting in a compatibility mismatch between the HLB = 12 emulsifier blend and the increasingly hydrophobic composite oil phase. This mismatch led to diminished molecular packing density at the oil–water interface, weakened interfacial stability, and subsequent droplet coalescence, ultimately giving rise to a pronounced increase in particle size [53,54]. Conversely, HLB = 13 and 14 formed extremely small, uniform droplets after OCT loading (10.37 ± 0.17 nm, PDI = 0.281 ± 0.014; 11.42 ± 0.37 nm, PDI = 0.244 ± 0.020, respectively), with no significant difference between them. This indicates that the emulsifiers at HLB = 13 and 14 exhibit stronger hydrophilicity, which not only maintains appropriate interactions with the oil phase but also forms stable hydrogen bonds with the aqueous phase. These properties sustain the interfacial characteristics favorable for generating smaller droplets, thereby effectively preserving the stability of the microemulsion [55]. Although OCT-ME particle sizes were similar at HLB = 13 and 14, the stable region at HLB = 13 was significantly larger. Furthermore, the HLB = 13 system exhibited excellent structural stability both before and after OCT addition. Collectively, the optimal HLB value for the emulsifier blend was determined to be 13.

#### 3.2.4. Determination of Co-Emulsifier

As shown in Figure 1G, the stable microemulsion region size varied markedly with short-chain alcohol type. Specifically, glycerol exhibited the largest microemulsion region (16.55 ± 0.02%), followed by 1,2-propanediol (12.38 ± 0.01%), whereas ethanol yielded the smallest region (10.37 ± 0.03%). These findings demonstrate a positive correlation between microemulsion region size and co-emulsifier carbon chain length. Short-chain alcohols with longer carbon chains promote preferential accumulation at the oil–water interface, enhancing interfacial film flexibility and dilution stability [56]. Accordingly, ethanol (shortest chain) showed the smallest area. Moreover, for co-emulsifiers with the same carbon chain length, a higher number of hydroxyl groups can significantly strengthen hydrogen bonding interactions, further loosening the interfacial film structure and lowering interfacial tension, thereby favoring the expansion of the stable microemulsion region [57].

As illustrated in Figure 1J, the variations in particle size and PDI of microemulsion systems prepared with different co-emulsifiers were compared before and after OCT incorporation. In the absence of OCT, the mean particle size of the ethanol system (35.57 ± 1.54 nm, PDI = 0.242 ± 0.054) was significantly larger than those of systems formulated with 1,2-propanediol (10.79 ± 0.05 nm, PDI = 0.249 ± 0.002) and glycerol (12.11 ± 0.02 nm, PDI = 0.363 ± 0.020). Upon OCT loading, the structural stabilities of the systems diverged markedly. The 1,2-propanediol-based system maintained virtually unchanged particle size and PDI (10.37 ± 0.17 nm and 0.281 ± 0.014, respectively), demonstrating excellent OCT compatibility and high structural robustness. In stark contrast, the particle size of the glycerol-based system surged from 12.11 ± 0.02 nm to 32.22 ± 0.64 nm, accompanied by a sharp increase in PDI to 0.961 ± 0.068, indicating near-complete destabilization and extensive droplet coalescence. Likewise, although the ethanol-based system showed only a slight increase in particle size (to 36.38 ± 1.09 nm), its PDI rose drastically from 0.242 ± 0.054 to 0.913 ± 0.150, signifying a breakdown in dispersion uniformity and severe intraparticle aggregation. The larger particle size in the glycerol system is attributed to its much higher viscosity, which hinders OCT diffusion, compromises interfacial film integrity, and promotes droplet coalescence [58]. In contrast, ethanol is overly water-soluble and partitions preferentially into the aqueous phase, resulting in insufficient interfacial flexibility and increased droplet coalescence [59]. Conversely, 1,2-propanediol displays favorable microemulsion-forming capacity while exhibiting a moderate viscosity close to that of the aqueous phase, which does not disturb the dispersion behavior of OCT in the oil phase. Consequently, it maintains exceptionally small and uniform droplet sizes both before and after OCT incorporation, affording the best overall performance [57]. Taken together, 1,2-propanediol was identified as the optimal co-surfactant for the preparation of the OCT-ME.

#### 3.2.5. Determination of the Km Value

The Km value is a critical parameter governing interfacial characteristics. At the optimal Km value, the co-emulsifier optimizes interfacial film flexibility and strength, promoting microemulsion formation and stability [60]. As illustrated in the pseudo-ternary phase diagram (Figure 1H), the extent of the stable microemulsion region varied markedly with the Km value. The microemulsion region reached its maximum (15.66 ± 0.01%) at Km = 5:1, followed by that at Km = 3:1 (12.29 ± 0.04%). In contrast, the stable region was significantly diminished at Km = 1:1 (4.88 ± 0.03%) and completely absent at Km = 1:3 (0.00 ± 0.00%). These results indicated that a higher proportion of emulsifier (higher Km) was generally conducive to expansion of the stable microemulsion region, which is consistent with the findings reported by Patel and Patel [61]. This phenomenon is attributed to the increased emulsifier content enhancing the formation and stability of the interfacial film, which consequently lowers interfacial tension, improves emulsification efficiency, and enlarges the stable microemulsion domain [62]. Conversely, high co-emulsifier proportions (lower Km) lead to a weakened interfacial film and a significant reduction or complete loss of the stable microemulsion region. This is due to their strong hydrophilicity causing excessive film stretching, and competitive adsorption by excess co-emulsifiers disrupting the ordered packing of emulsifiers [63].

To further evaluate the OCT loading capacity and stability of the formulations, changes in particle size and PDI were compared among systems with different Km values before and after OCT incorporation (Figure 1K). In the absence of OCT, systems with high emulsifier proportions (Km = 5:1 and 3:1) formed extremely small droplets, with mean particle sizes of 8.72 ± 0.12 nm (PDI = 0.237 ± 0.016) and 10.79 ± 0.05 nm (PDI = 0.249 ± 0.002), respectively. In contrast, the initial particle sizes of systems with low emulsifier proportions (Km = 1:1 and 1:3) reached 130.90 ± 0.82 nm (PDI = 0.437 ± 0.014) and 216.73 ± 3.77 nm (PDI = 0.393 ± 0.025), respectively, indicating that insufficient emulsifier content hindered the formation of a compact interfacial film. Upon OCT incorporation, marked differences in stability were observed among all systems. For the Km = 1:1 and 1:3 systems, the mean particle size increased sharply to 205.10 ± 7.05 nm (PDI = 0.880 ± 0.208) and 241.83 ± 12.56 nm (PDI = 0.857 ± 0.248), respectively, accompanied by a significant increase in PDI to above 0.8, indicating severe droplet coalescence. This phenomenon can be primarily attributed to the insufficient emulsifier content, which failed to form a compact interfacial film to encapsulate the highly hydrophobic OCT molecules, ultimately resulting in complete destabilization of the systems [64]. In comparison, both the Km = 5:1 and Km = 3:1 formulations yielded optically transparent OCT-MEs after OCT loading, yet marked differences were evident in their OCT loading adaptability. Although Km = 5:1 had the largest microemulsion region and smallest initial particle size, its size increased to 12.28 ± 0.42 nm, and PDI rose to 0.389 ± 0.014 after OCT addition. By contrast, the Km = 3:1 system displayed no particle size enlargement upon OCT incorporation; instead, the particle size decreased slightly to 10.37 ± 0.17 nm while retaining a low and uniform PDI of 0.281 ± 0.014. This phenomenon can mainly be ascribed to the long carbon chain and strong lipophilicity of OCT, which strengthen attractive interactions between droplets, trigger partial particle aggregation, and thus adversely affect the stability of the OCT-MEs. Relative to the Km = 5:1 system, the Km = 3:1 formulation exhibited a higher co-emulsifier proportion, which effectively counteracted the enhanced inter-droplet attraction induced by OCT, resulting in a more homogeneous system dispersion [65]. Furthermore, higher emulsifier dosages increase production costs, and excess Tween 80/Span 80 may impair product sensory properties (aroma and taste). Therefore, despite the larger stable microemulsion region observed in the Km = 5:1 system, the Km = 3:1 formulation exhibited superior stability after OCT loading, with a more appropriate dosage that balanced economic viability and practical sensory requirements. Collectively, a Km value of 3:1 was selected as the optimal condition in this study.

### 3.3. Determination of OCT Loading

Based on the optimized formulation established in Section 3.2, the effect of OCT loading content (0.0–1.2%, *w*/*w*) on the particle size and PDI of OCT-ME was investigated to determine the optimal loading level. As shown in Figure 1I,L, increasing the OCT loading from 0% to 0.2% resulted in a significant decrease in mean particle size from 11.85 ± 0.08 nm to 10.31 ± 0.04 nm, accompanied by a reduction in PDI from 0.365 ± 0.006 to 0.255 ± 0.006. These results suggest that an appropriate amount of OCT can be efficiently incorporated into the oil phase, yielding a more compact internal structure and enhanced dispersion homogeneity. With a further increase in OCT loading to 0.4–1.0%, the particle size remained relatively stable within a narrow range of 10.26–10.47 nm, while PDI values were maintained at a low level of approximately 0.3. These results indicate that, within this loading range, the microemulsion micelles exhibited sufficient solubilization capacity for OCT, enabling the system to maintain favorable thermodynamic stability. In contrast, when the OCT loading reached 1.2%, a sharp increase in particle size (16.42 ± 0.20 nm) and a marked elevation in PDI (0.779 ± 0.015) were observed, both of which were significantly higher than those of the other groups, indicating severe destabilization of the microemulsion system. This instability can be ascribed to the OCT loading exceeding the critical solubilization capacity of the micelles, such that excess OCT could no longer be effectively encapsulated, thereby inducing crystallization or droplet coalescence and ultimately leading to system collapse [66]. Considering both loading efficiency and colloidal stability, the OCT-ME with an OCT loading of 1.0% exhibited a favorable balance between relatively high OCT content, small particle size (10.42 ± 0.14 nm), and good dispersion uniformity (PDI = 0.295 ± 0.004). Accordingly, an OCT loading of 1.0% was selected as the optimal level for subsequent experiments.

Based on the comprehensive optimization of all formulation parameters, the final optimal food-grade OCT-ME was determined to contain 1.0% (*w*/*w*) OCT, 2.4% (*w*/*w*) corn oil, 13.3% (*w*/*w*) Tween 80, 2.9% (*w*/*w*) Span 80, 5.4% (*w*/*w*) 1,2-propanediol (Km = 3:1), and 75.0% (*w*/*w*) deionized water. All subsequent characterization experiments were conducted using this standardized formulation.

### 3.4. Configuration Analysis of OCT-ME

**Figure 2:**
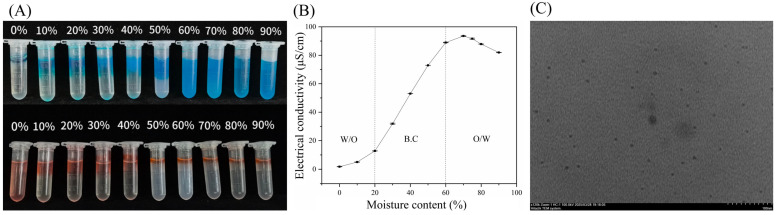
Diffusion of different dyes in OCT-ME with different water contents (**A**); conductivity changes in OCT-ME with different water contents (**B**); transmission electron microscopy (TEM) images (**C**).

The staining method distinguishes phase configurations by monitoring the diffusion of hydrophilic dye (methylene blue, MB) and lipophilic dye (Sudan Red, SR) in OCT-ME at different water mass fractions (Figure 2A). When the water content was below 30% (*w*/*w*), the MB solution was confined exclusively to the upper region of the system with no internal diffusion, whereas the SR solution was dispersed uniformly throughout the entire system. This observation indicated that the microemulsion was of the water-in-oil (W/O) type. As the water content increased to 30–60% (*w*/*w*), MB diffused considerably faster and across a progressively larger region, suggesting a phase transition from W/O toward a bicontinuous structure, wherein the oil and aqueous phases gradually formed interconnected continuous domains. When the water content exceeded 60% (*w*/*w*), SR accumulated near the top, whereas MB spread rapidly and became uniformly distributed, corresponding to a stable O/W ME. This evolution is consistent with previous reports on microemulsion structural transitions for hydrophobic actives such as carvacrol and Forsythia essential oil [67,68].

In this study, electrical conductivity measurements were performed to further validate the structural transition of OCT-ME (Figure 2B). At water contents below 30% (*w*/*w*), the conductivity increased slowly, from 1.76 ± 0.10 to 12.77 ± 0.07 μS/cm, consistent with a W/O structure. As water content increased, emulsifier dissociation promoted a rise in conductivity; however, the oil-continuous phase constrained the rate of this increase [69]. In the 30–60% (*w*/*w*) water content range, the conductivity increased sharply from 31.90 ± 0.09 to 88.95 ± 0.21 μS/cm. This pronounced increase signifies the formation of interconnected bicontinuous channels between the oil and water phases, wherein reverse micellar droplets form conductive pathways through collisions, thereby facilitating ion migration and signaling the phase transition from W/O to a bicontinuous structure. This observation is consistent with the conductivity evolution patterns reported in previous studies [70]. At water contents above 60% (*w*/*w*), the increase in conductivity became less pronounced, reaching a maximum of 93.55 ± 0.17 μS/cm at 70% (*w*/*w*) followed by a slight decrease, suggesting that the system had stabilized as an O/W ME. The elevated water content strengthened interactions between the co-emulsifier and water molecules, with most water molecules binding to polyols, leading to micellar swelling and a reduced micellar number density, which consequently led to a decrease in conductivity [71,72]. Overall, the conductivity results corroborate the dye-staining observations, confirming a sequential W/O–bicontinuous–O/W transition with increasing water content. The formulation employed in this study, containing 75% (*w*/*w*) water, lies within the stable O/W ME region. It exhibits a conductivity of 91.67 ± 0.43 μS/cm, indicating that the aqueous phase forms a stable conductive network. Meanwhile, the system demonstrates favorable OCT loading efficiency and stability, rendering it suitable for subsequent application studies.

### 3.5. Transmission Electron Microscopy (TEM) Analysis

Figure 2C (100 nm) and Figure S2 (200 nm) show the microstructural characteristics of OCT-ME at different magnifications. The droplets displayed a well-defined spherical morphology with a relatively narrow size distribution, and most were smaller than 50 nm with no obvious aggregation. These observations indicated that OCT was efficiently incorporated into the microemulsion system, and the as-prepared OCT-ME exhibited favorable dispersibility and stability. Furthermore, the TEM findings were in agreement with the DLS data, further verifying the successful formation of OCT-ME.

### 3.6. Rheological Properties Analysis

**Figure 3:**
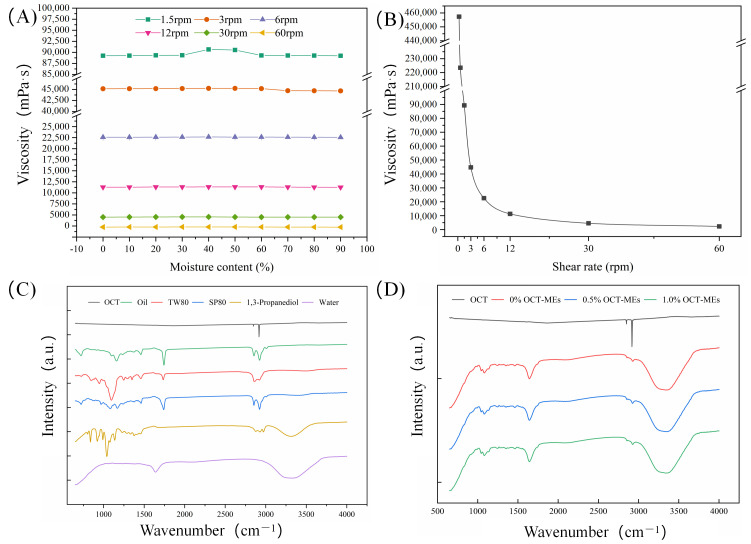
Viscosity of OCT-MEs with different water contents at different shear rates (**A**); viscosity of OCT-ME with 75% water content at different rotation speeds (**B**); Fourier transform infrared (FT-IR) spectra of OCT and other microemulsion components (**C**); FT-IR spectra of OCT-ME at different OCT loadings (**D**).

This study elucidated the rheological behavior of OCT-ME by investigating the effects of water content and shear rate on viscosity. As shown in Figure 3A, at a fixed shear rate, viscosity remained relatively stable over the water content range of 0–90% (*w*/*w*), with only minor fluctuations. At the most pronounced fluctuation point (1.5 rpm), viscosity varied from 89,244 ± 2.65 to 89,347 ± 3.61 mPa·s, corresponding to a fluctuation range less than 2%. A slight viscosity increase was observed in the 40–60% (*w*/*w*) range, attributed to the formation of an ordered bicontinuous structure [73]. This unique structure imparts increased viscoelasticity to the system, resulting in a moderate increase in viscosity. The viscosity–shear-rate relationship was investigated (Figure 3B). The system exhibited typical shear-thinning behavior: at a low shear rate of 0.3 rpm, the viscosity reached as high as 457,283 ± 33 mPa·s and decreased markedly with increasing shear rate, dropping to 2255 ± 2 mPa·s at 60 rpm. On a log–log scale, the viscosity–shear rate profile was well-fitted by the power-law model, $\eta =K\gamma^{\cdot n-1}(n<1)$, confirming that the system behaves as a non-Newtonian pseudoplastic fluid [74]. The observed viscosity variations are primarily ascribed to the shear-induced disruption and reorientation of the internal microstructure and micellar networks. This behavior ensures structural integrity and stable dispersion during storage at low shear rates, while facilitating processing and mixing operations by reducing apparent viscosity at high shear rates [75]. Accordingly, the shear-thinning behavior of OCT-ME is highly favorable for its processing and application in the food industry.

### 3.7. FT-IR Analysis

As shown in Figure 3C, each component of OCT-ME exhibited its characteristic FT-IR peaks. For corn oil, Tween 80, and Span 80, the absorption peaks at 2924 cm^−1^ and 2854 cm^−1^ are assigned to the asymmetric and symmetric stretching vibrations of CH_2_ groups, respectively. Furthermore, the absorption peak near 1741 cm^−1^ corresponds to the stretching vibration of C = O bonds, a characteristic peak of oils and emulsifiers. The region 3100–3600 cm^−1^ exhibits broad and intense O–H stretching bands of 1,2-propanediol and water. Among these, the absorption peak of 1,2-propanediol at approximately 1182 cm^−1^ is attributed to the stretching vibration of C–O bonds. In contrast, OCT exhibited relatively weak absorption peaks at 2916 cm^−1^ and 2848 cm^−1^, assigned to the asymmetric and symmetric stretching vibrations of CH_2_ groups in its long alkyl chain. Notably, no distinct O–H stretching vibration peak of OCT was observed. This phenomenon is likely attributed to the ordered crystalline packing of solid OCT, which restricts hydrogen bond formation and consequently weakens the intensity of the O–H stretching band [76].

As presented in Figure 3D, the characteristic absorption peaks of all components were preserved in the FT-IR spectrum of OCT-ME, including those of C–O (≈1080 cm^−1^), C = O (≈1668 cm^−1^), CH_2_ (≈2924 cm^−1^ and 2854 cm^−1^), and O–H (≈3332 cm^−1^). Among these peaks, the O–H band was significantly broadened and intensified, indicating enhanced hydrogen bonding between water, 1,2-propanediol, and emulsifiers [77]. Notably, the CH_2_ stretching peaks of OCT at 2916 cm^−1^ and 2848 cm^−1^ were markedly attenuated, indicating that solubilization of OCT within the microemulsion masks its molecular signals in the emulsion matrix [76]. Furthermore, the FT-IR spectra of OCT-MEs with various OCT loadings overlapped almost completely. This result further confirms that OCT was fully dispersed either in the oil core or at the oil–water interface of the microemulsion, with its characteristic FT-IR signals shielded by the surrounding medium. Importantly, the solubilization of OCT did not induce appreciable changes in the microemulsion microstructure or in the intermolecular interactions among its constituents, which was consistent with the spectral behavior of bioactive components encapsulated in the microemulsion cores, as documented in previous studies [78]. Collectively, these FT-IR results provide molecular-level evidence that OCT can be stably dispersed within OCT-ME, thereby underpinning its potential as an effective functional delivery system.

### 3.8. Stability Study of OCT-ME

The stability of OCT-ME directly determines the shelf life, functionality, and safety of food products [37]. Given that centrifugal stress, temperature fluctuations, pH and salinity variations, and prolonged storage can compromise OCT-ME stability in practical applications, this study systematically monitored variations in particle size, PDI, and macroscopic appearance under the aforementioned conditions to comprehensively evaluate its stability.

#### 3.8.1. Centrifugal Stability

**Figure 4:**
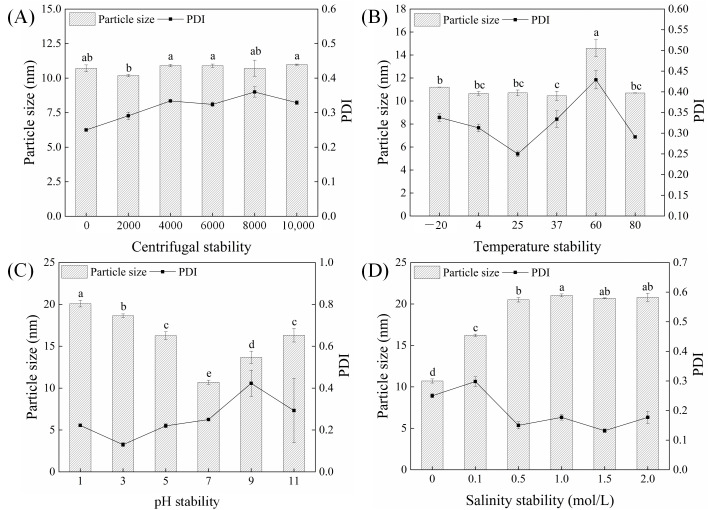
Changes in particle size and PDI of OCT-ME under different conditions: centrifugation (**A**); temperature (**B**); pH (**C**); salinity (**D**). Different lowercase letters within the same group indicate significant differences (*p* < 0.05).

As shown in Figure 4A and Figure S3A, OCT-ME exhibited no visually observable phase separation or precipitation over the centrifugal range of 0–10,000 rpm. The mean diameter increased slightly from 10.19 ± 0.25 nm to 10.98 ± 0.15 nm, with no significant difference (*p* > 0.05) relative to the initial sample. The PDI ranged from 0.250 to 0.360, indicating a relatively narrow and uniform particle size distribution. These results indicate that the OCT-ME interfacial film has high mechanical strength and anti-aggregation capability, ensuring excellent stability at high centrifugal speeds.

#### 3.8.2. Temperature Stability

As shown in Figure 4B and Figure S3B, OCT-ME maintained a homogeneous and transparent appearance over the range of −20 to 80 °C, with no visible phase separation or precipitation. Using the sample at 25 °C as the reference (particle size: 10.71 ± 0.25 nm, PDI: 0.250 ± 0.007), the droplets maintained excellent stability at −20 °C, 4 °C, 37 °C, and 80 °C, with particle size ranging from 10.45 ± 0.40 nm to 11.21 ± 0.01 nm and PDIs between 0.250 and 0.338, showing no significant differences relative to the initial sample. Notably, at 60 °C, the particle size and PDI increased markedly to 14.60 ± 0.74 nm and 0.429, respectively, which can be attributed to the system approaching the phase inversion temperature of the emulsifier blend. At this temperature, the emulsifier polar head group dehydration increases lipophilicity, weakens interfacial stability, and promotes droplet coalescence, reducing overall stability [79]. Overall, OCT-ME shows excellent stability at most temperatures, with only slight coalescence at 60 °C and no irreversible phase separation. This makes it suitable for cold-chain logistics, ambient transportation and moderate thermal processing in the food industry.

#### 3.8.3. pH Stability

As shown in Figure 4C and Figure S3C, under strongly acidic conditions (pH 1), the particle size reached a maximum of 20.09 ± 0.40 nm with a PDI of 0.222 ± 0.007. At pH 7, OCT-ME exhibited optimal stability, with the particle size decreasing to a minimum value (10.71 ± 0.25 nm) and a PDI of 0.250 ± 0.007. This result indicated that a neutral environment is more conducive to maintaining the structural integrity of the interfacial film and the anti-coalescence capacity of OCT-ME. However, as the pH value further increased to the alkaline range (pH 9 and 11), the particle sizes rose again to 13.68 ± 0.74 nm and 16.32 ± 0.81 nm, with corresponding PDIs of 0.423 ± 0.063 and 0.293 ± 0.154, respectively, suggesting a significant reduction in system stability. This behavior may be attributed to the influence of strongly acidic or alkaline environments on the aggregation behavior and solvation characteristics of the emulsifiers, thereby altering the interfacial tension and facilitating droplet coalescence [80]. Overall, OCT-ME exhibited a certain tolerance to acidic and alkaline environments and showed excellent pH stability under neutral conditions. Even at extreme pH values, no irreversible phase separation occurred, making it applicable in diverse scenarios such as acidic beverages, neutral dairy products, and gastrointestinal digestion. Therefore, in practical applications, product pH can be adjusted as needed to maintain OCT-ME’s optimal stable state.

#### 3.8.4. Salinity Stability

As shown in Figure 4D and Figure S3D, the particle size increased from the initial 10.71 ± 0.25 nm to 16.22 ± 0.15 nm at 0.1 mol/L and further to 20.51 ± 0.27 nm at 0.5 mol/L. This increase in particle size can be attributed to the high-salt-induced shielding effect, compressing the electrical double layer, weakening inter-droplet electrostatic repulsion, and promoting reversible aggregation [81]. At NaCl concentrations ranging from 0.5 to 2.0 mol/L, the particle size remained between 20.51 ± 0.27 and 21.04 ± 0.17 nm, with no significant differences (*p* > 0.05) observed, indicating that the droplets achieved a relatively stable size under elevated salinity conditions. Overall, OCT-ME exhibited robust stability at low to moderate salt concentrations (0–1.5 mol/L NaCl) and is suitable for most salt-containing food matrices (e.g., beverages and soups). For high-salt food products, formulation optimization or the incorporation of stabilizers may be required to maintain its stable performance.

#### 3.8.5. Storage Stability

**Figure 5:**
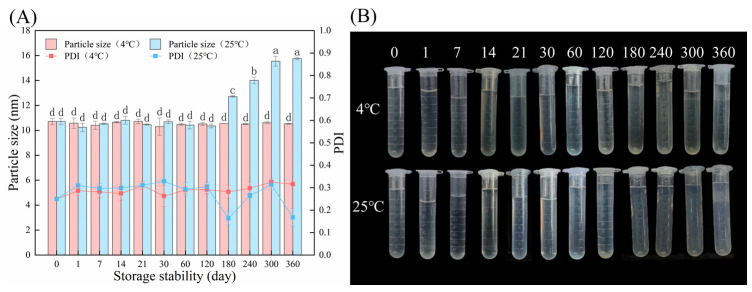
Changes in particle size, PDI (**A**), and appearance (**B**) of OCT-ME under long-term storage. Different lowercase letters within the same group indicate significant differences (*p* < 0.05).

As shown in Figure 5A,B, no precipitation or phase separation occurred at 4 °C or 25 °C over 360 days, and the system remained uniformly transparent. At 4 °C, the mean particle size of OCT-ME remained stable in the range of 10.31 ± 0.69 nm to 10.72 ± 0.18 nm throughout the storage period, with no significant difference compared with the initial value (*p* > 0.05). The PDI fluctuated slightly within the range of 0.250 ± 0.007 to 0.316 ± 0.002, indicating excellent homogeneity. In contrast, at 25 °C (room temperature), the particle size of OCT-ME showed a distinct temporal evolution pattern over the storage period. During the first 120 days, the particle size fluctuated slightly within the range of 10.24 ± 0.33 nm to 10.80 ± 0.31 nm, with no significant difference relative to the initial value (*p* > 0.05). From day 120 to day 300, the mean particle size of OCT-ME showed a continuous upward trend with prolonged storage, increasing significantly from 10.36 ± 0.15 nm to 15.55 ± 0.40 nm (*p* < 0.05). Subsequently, from day 300 to day 360, the particle size tended to stabilize and finally plateaued at 15.76 ± 0.10 nm, showing no significant difference compared with the value on day 300 (*p* > 0.05). The PDI fluctuated slightly throughout the storage period but consistently remained below 0.350, with no discernible trend of deterioration.

The difference in stability between the two temperatures can be attributed to the following mechanisms. At 4 °C, low temperature suppresses the Brownian motion of droplets and weakens intermolecular interactions, thereby preventing irreversible aggregation and maintaining the initial particle size and dispersion uniformity of the system [82]. At 25 °C, higher temperature increases droplet diffusion and solubility, accelerates Ostwald ripening, reduces viscosity, and increases droplet collision frequency, leading to aggregation and particle size increase [83,84]. Nevertheless, the densely packed interfacial film of OCT-ME provides sufficient mechanical strength to resist further coalescence of droplets; thus, the particle size tended to be stable in the later storage stage [85]. Collectively, OCT-ME exhibits excellent long-term storage stability at 4 °C. Although a certain degree of particle size increase was observed at 25 °C, it stabilized in late storage, with no precipitation or irreversible phase separation. These results indicate that OCT-ME possesses favorable storage stability spanning from refrigeration to room temperature, thereby providing a reliable technical basis for its application in food products under diverse storage conditions.

### 3.9. In Vitro Digestion Characteristics of OCT-ME

**Figure 6:**
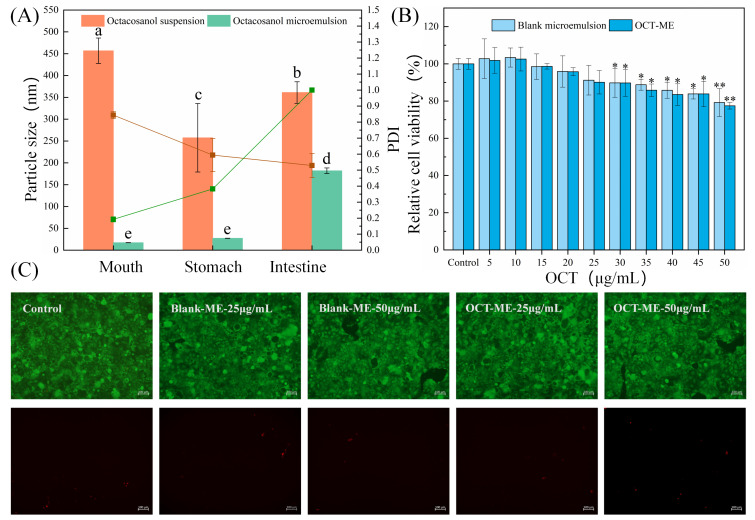
(**A**) Particle size and PDI of OCT suspension and OCT-ME during in vitro simulated oral, gastric and intestinal digestion; (**B**) relative viability of Caco-2 cells treated with blank microemulsion and OCT-ME for 24 h; (**C**) live/dead fluorescence staining images of Caco-2 cells after 24 h treatment with blank microemulsion and OCT-ME (green: live cells, red: dead cells). Different lowercase letters within the same group indicate significant differences (*p* < 0.05). * *p* < 0.05 and ** *p* < 0.01 compared with the control group.

In this study, simulated oral, gastric, and intestinal digestion models were used to compare OCT-ME and OCT suspensions with respect to particle size, PDI, and OCT retention under the corresponding digestive conditions (Figure 6A; Table 2), so as to systematically evaluate the stability of the ME-based delivery system and the release behavior of OCT. During the oral digestion stage, the OCT-ME maintained a small initial particle size (17.18 ± 0.29 nm) and a low PDI (0.193 ± 0.016), whereas the OCT suspension formed significantly larger particles (465.30 ± 24.50 nm) with a higher PDI (0.748 ± 0.095), indicating the superior initial dispersion stability of the ME-based delivery system. Meanwhile, the OCT retention of both delivery systems exceeded 98%, which was attributed to the absence of specific lipolytic enzymes in the oral environment, as well as no significant alterations in the pH and ionic strength [86].

During the gastric digestion stage, the microemulsion maintained nanosized droplets (27.28 ± 0.26 nm) with a low PDI (0.383 ± 0.007), whereas the particle size of the suspension decreased to 245.77 ± 8.96 nm, accompanied by a reduction in PDI to 0.608 ± 0.109. Meanwhile, the OCT retention of the suspension decreased markedly to 33.60 ± 0.39%, whereas the microemulsion maintained a relatively high retention of 54.81 ± 2.20%. These results suggest that the stable structure of the microemulsion remained intact under gastric conditions, as its dense interfacial film effectively encapsulated OCT, reduced direct contact with the gastric environment, and consequently minimized its degradation [87]. In contrast, although protonation of the emulsifier in the suspension enhanced its interfacial adsorption capacity and alleviated particle aggregation, leading to a slight reduction in particle size, the system still exhibited poor stability with limited interfacial protection. As a result, OCT was released rapidly and extensively, resulting in a significantly lower retention rate [88].

Upon intestinal digestion, the OCT retention for both delivery systems decreased significantly, reaching 8.21 ± 0.06% for the microemulsion and 16.20 ± 0.05% for the suspension, respectively. Simultaneously, the particle size of OCT-ME increased sharply to 141.53 ± 18.55 nm, yet remained considerably smaller than that of the suspension (361.10 ± 24.61 nm), and PDI increased to 0.606 ± 0.163, which was slightly higher than that of the suspension (0.529 ± 0.075). This phenomenon is closely associated with interfacial remodeling induced by bile salts, phospholipids, and pancreatic lipase in the intestinal milieu [89]. On one hand, bile salts compete with emulsifiers for adsorption, weakening the interfacial film and triggering reversible aggregation or mild coalescence of oil droplets, as reflected by the increase in particle size [90]. On the other hand, mixed micelles formed by bile salts and emulsifiers exhibit a superior solubilization capacity toward OCT, promoting its translocation from oil droplets into the aqueous micellar phase and thus resulting in a decrease in retention rate. However, the increases in particle size and decreases in retention rate observed in the intestinal phase do not signify instability or compromised protection of the microemulsion. Instead, these changes largely indicate that OCT is released and partitions into a more readily absorbable micellar phase, which is favorable for enhancing the oral bioavailability of OCT [91]. In contrast, although the suspension exhibited a higher OCT retention rate, its large particle size and inferior dispersibility impeded effective solubilization by bile salts. This suppressed OCT release and limited its intestinal bioaccessibility, which is consistent with the findings of Deguchi et al. [92]. Collectively, OCT-ME demonstrates superior stability and controlled release behavior during in vitro digestion, effectively enhancing the bioavailability of OCT while showing considerable promise for applications in functional foods.

### 3.10. Biocompatibility Analysis of OCT-ME

To evaluate the biocompatibility of the as-prepared OCT-ME as an oral delivery system, the CCK-8 assay was used to quantitatively assess the effects of blank microemulsions and OCT-ME at different concentrations (5–50 μg/mL) on Caco-2 cell viability (Figure 6B).

At 5–25 μg/mL, the cell viability for both formulations remained at approximately or above 90% relative to the control, with no significant differences (*p* > 0.05), indicating that the emulsifier system exhibits excellent biocompatibility within this concentration range and that OCT loading did not introduce additional cytotoxicity risk. More importantly, previous studies have shown that daily intake of 10–20 mg OCT can exert physiological effects, including anti-fatigue, anti-hypoxia, cholesterol-lowering, and enhancement of physical strength and endurance [93,94,95]. For a 500 mL functional beverage, only 1–2 mL of the prepared OCT-ME is required to achieve the desired efficacy, corresponding to a concentration of approximately 10–20 μg/mL, which is well within the safe range verified in this experiment and fully meets the practical application requirements of functional foods.

When the concentration increased to 30–50 μg/mL, the cell viability showed a concentration-dependent decrease, with a significant difference compared with the control group (*p* < 0.05). Furthermore, starting from 35 μg/mL, the viability of the OCT-ME group was slightly lower than that of the blank microemulsion group. This may be attributed to OCT, a lipophilic long-chain fatty alcohol, which interacts with the cellular phospholipid bilayer through hydrophobic interactions, thereby affecting membrane fluidity and selective permeability and further imposing a mild metabolic burden and energy consumption pressure on cells, leading to decreased viability [96,97]. Notably, even at the concentration of 50 μg/mL, the cell viability remained above 75%, with no obvious acute cytotoxicity, which meets the safety requirements for oral delivery systems. This result is consistent with the findings of Gao et al. [98] on LRE microemulsions.

To further verify the biocompatibility of OCT-ME, a dual fluorescence staining assay was performed to visually evaluate the membrane integrity of Caco-2 cells. As shown in Figure 6C, the control group exhibited intense green fluorescence (viable cells) and very weak red fluorescence (dead cells), indicating a good cellular state. At 25 μg/mL, the fluorescence performance of both blank microemulsions and OCT-ME was not significantly different from that of the control group: the green fluorescence intensity was high, cells covered the entire field of view with intact morphology, and red fluorescence was barely detectable, indicating good cell membrane integrity and vigorous metabolic activity. At 50 μg/mL, green fluorescence intensity and cell density slightly decreased in both groups, but no widespread necrosis or morphological distortion was observed, only a few scattered red fluorescence spots, indicating minimal membrane damage with viable cells still predominant. The above results are consistent with those obtained by the CCK-8 assay.

Collectively, the results of cytotoxicity and morphological evaluation indicate that OCT-ME has no obvious cytotoxicity within the concentration range of 5–25 μg/mL, with high cell viability and intact cell morphology, and meets the practical application concentration requirements of functional beverages, which can be used as a safe reference range for its application in food systems. Even at concentrations much higher than the daily addition amount, OCT-ME did not show obvious acute cytotoxicity and had good overall biocompatibility, providing reliable safety support for its application in the functional food and pharmaceutical fields.

## 4. Conclusions

In this study, a food-grade OCT-ME with a high loading capacity of 1.0% (*w*/*w*) was successfully developed, which markedly outperforms the previously reported OCT loading levels ranging from 0.1% to 0.55% (*w*/*w*). This system maintained excellent stability for up to 360 days during storage at 4 °C and also exhibited robust tolerance to multiple environmental stresses, including centrifugation, thermal fluctuations, wide pH variations, and high salinity conditions. In vitro simulated gastrointestinal digestion assays revealed that the OCT-ME could effectively shield OCT from degradation in the gastric phase while facilitating its targeted release in the intestinal phase, thus significantly enhancing its oral bioavailability. Caco-2 cell cytotoxicity assays further confirmed the superior biocompatibility of the fabricated OCT-ME, with cell viability remaining above 90% at OCT concentrations of 5–25 μg/mL. Collectively, this study not only addresses the long-standing critical limitations of conventional OCT-ME systems, namely low loading capacity and excessive dosage requirements of emulsifiers and co-emulsifiers, but also provides a robust theoretical framework and practical technical route for the efficient, stable, and safe application of OCT and other lipophilic bioactive ingredients in functional beverage formulations.

## Figures and Tables

**Table 1:** Solubility of octacosanol (OCT) in the different oil phases.

Oil Phase	Solubility (mg/L)
Peanut oil	49.07 ± 0.07 ^a^
Coconut oil	46.32 ± 0.07 ^b^
Corn oil	39.67 ± 0.08 ^c^
Soybean oil	37.67 ± 0.10 ^d^
Olive oil	31.38 ± 0.24 ^e^

Values are expressed as mean ± standard deviation (n = 3). Different lowercase letters within the same column indicate significant differences (*p* < 0.05).

**Table 2:** Retention rates of OCT suspension/microemulsion during simulated in vitro oral, gastric, and intestinal digestion.

Sample	OCT Retention Rate (%)		
	Oral	Gastric	Intestinal
OCT suspension	98.04 ± 0.56	33.60 ± 0.39	16.20 ± 0.05
OCT microemulsion	99.72 ± 0.17	54.81 ± 2.20	8.21 ± 0.06

## Data Availability

The original contributions presented in this study are included in the article/Supplementary Material. Further inquiries can be directed to the corresponding author.

## Supplementary Materials

The following supporting information can be downloaded at: https://www.mdpi.com/article/10.3390/foods15122154/s1, Figure S1: Schematic Pseudoternary Phase Diagram of the OCT-ME Formulation System; Table S1: Formulations of Simulated Digestive Fluid Stock Solutions; Figure S2: Particle Morphology of the Optimal OCT-ME Characterized by Transmission Electron Microscopy (TEM); Figure S3: Appearance of OCT-ME under Different Conditions: Centrifugation (A); Temperature (B); pH (C); Salinity (D).

## Abbreviations

The following abbreviations are used in this manuscript:

OCT	Octacosanol
O/W ME	Oil-in-Water Microemulsion
OCT-ME	Octacosanol-loaded Microemulsion
HLB	Hydrophilic-Lipophilic Balance
PDI	Polydispersity Index
FT-IR	Fourier Transform Infrared Spectroscopy
GC-MS	Gas Chromatography-Mass Spectrometry
SSF	Simulated Saliva Fluid
SGF	Simulated Gastric Fluid
SIF	Simulated Intestinal Fluid
Smix	emulsifier and co-emulsifier mixture
TEM	Transmission Electron Microscopy
