# Supplementary material for: Food-Grade Microemulsion for High-Loading Octacosanol: Formulation Optimization, Characterization, and Biological Evaluation
Source: Foods. 2026 Jun 15;15(12):2154. doi: 10.3390/foods15122154 (PMC13298835; doi:10.3390/foods15122154)
Supplement: Supplementary file 1 [file foods-15-02154-s001.zip › Supplementary Material.pdf]

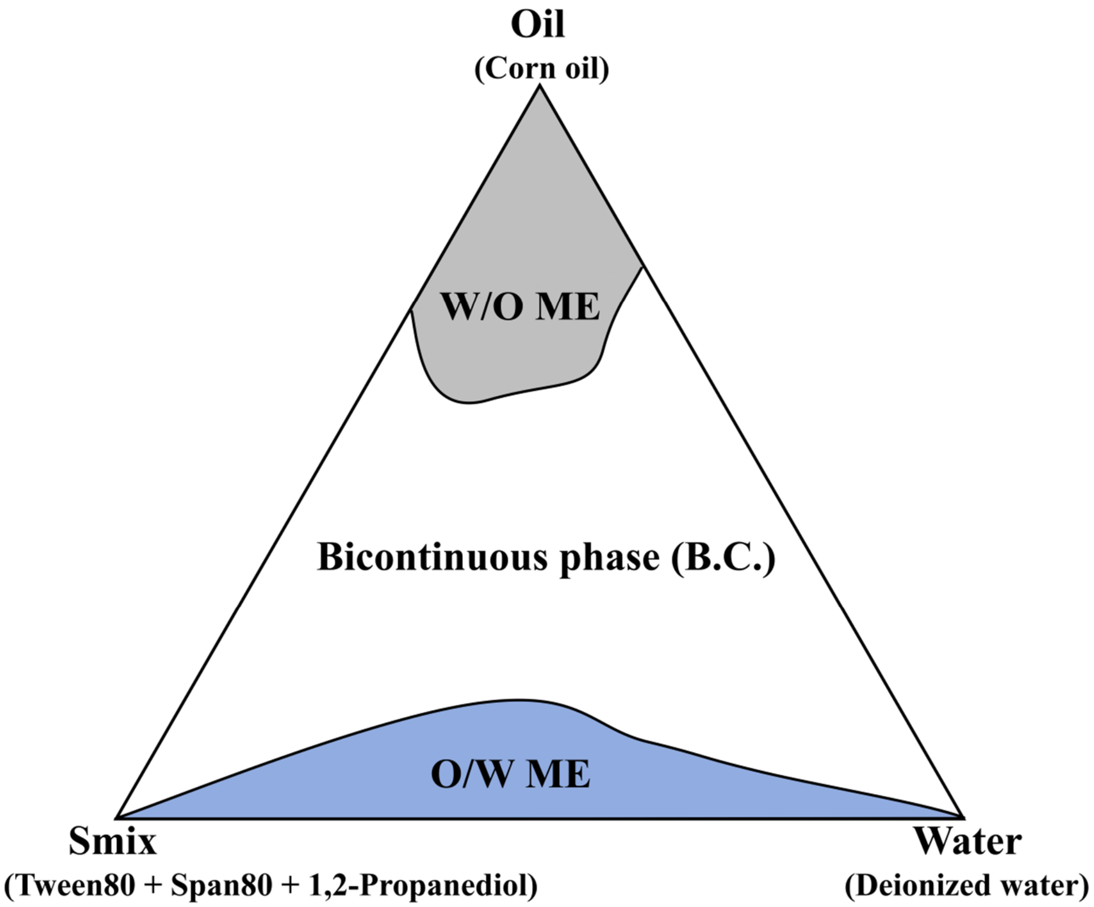

Figure S1. Schematic Pseudoternary Phase Diagram of the OCT-ME Formulation System

Table S1. Formulations of Simulated Digestive Fluid Stock Solutions.

Ingredients	Concentration(M)	SSF (mL)	SGF (mL)	SIF (mL)
KCl	0.50	3.775	1.725	1.700
KH_2PO_4	0.50	0.925	0.225	0.200
$NaHCO_3$	1.00	1.700	3.125	10.625
NaCl	2.00	—	2.950	2.400
$MgCl_2{\cdot}6H_2O$	0.15	0.125	0.100	0.275
$(NH_4)_2CO_3$	0.50	0.023	0.125	—
HCl	6.00	0.023	0.325	0.175
$CaCl_2{\cdot}2H_2O$(In addition)	0.30	0.025	0.005	0.040

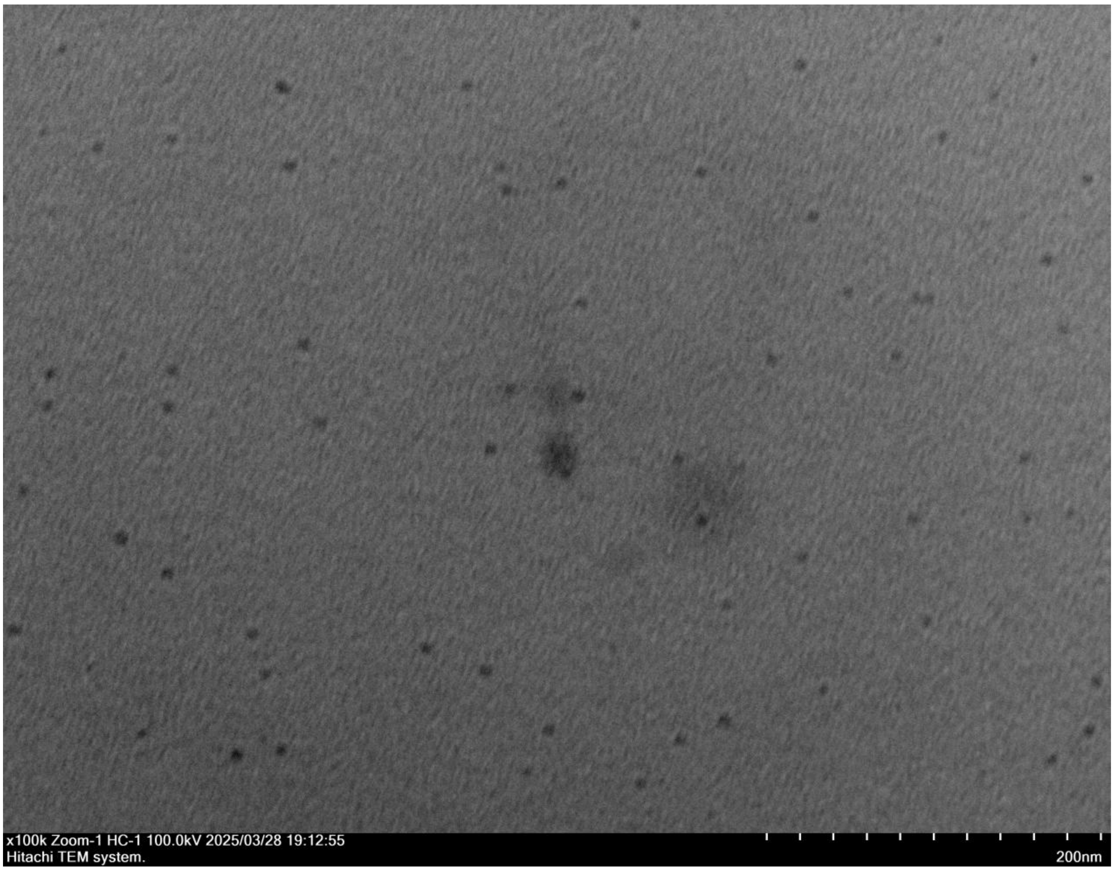

Figure S2. Particle morphology of the optimal OCT-ME characterized by transmission electron microscopy (TEM)

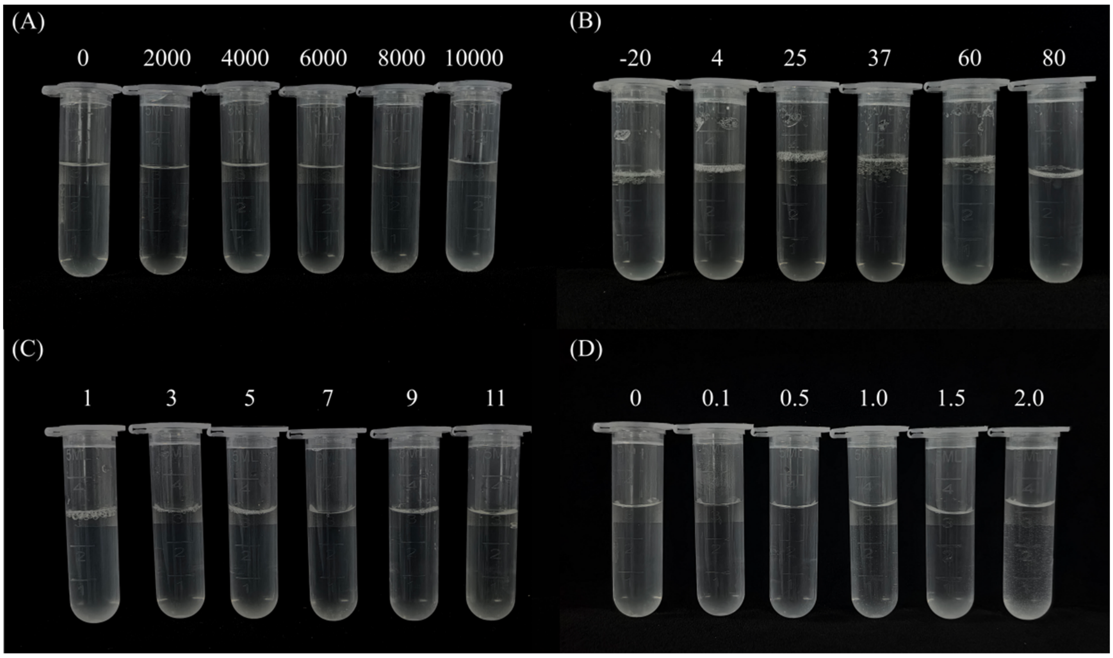

Figure S3. Appearance of OCT-ME under different conditions: Centrifugation (A); Temperature (B); pH (C); Salinity (D).
