# Supplementary figures and images for: Food-Grade Microemulsion for High-Loading Octacosanol: Formulation Optimization, Characterization, and Biological Evaluation
Source: Foods. 2026 Jun 15;15(12):2154. doi: 10.3390/foods15122154 (PMC13298835; doi:10.3390/foods15122154)

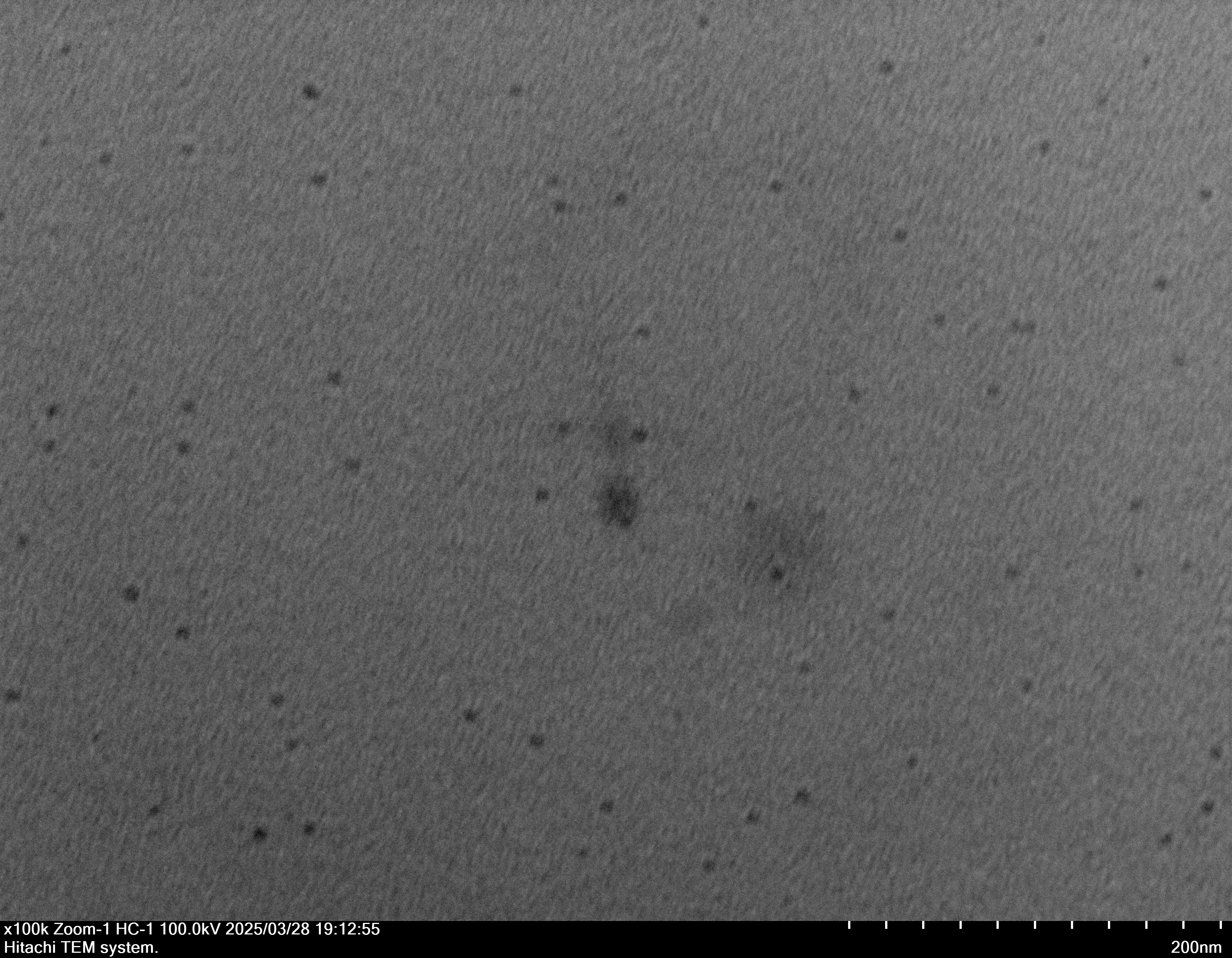

Supplement: Supplementary file 1 [file foods-15-02154-s001.zip › Figure S2.jpg]
